# Essential Oil of *Cymbopogon nardus* (L.) Rendle: A Strategy to Combat Fungal Infections Caused by *Candida* Species

**DOI:** 10.3390/ijms17081252

**Published:** 2016-08-09

**Authors:** Luciani Gaspar De Toledo, Matheus Aparecido Dos Santos Ramos, Larissa Spósito, Elza Maria Castilho, Fernando Rogério Pavan, Érica De Oliveira Lopes, Guilherme Julião Zocolo, Francisca Aliny Nunes Silva, Tigressa Helena Soares, André Gonzaga dos Santos, Taís Maria Bauab, Margarete Teresa Gottardo De Almeida

**Affiliations:** 1 Department of Biological Sciences, School of Pharmaceutical Sciences, Universidade Estadual Paulista, Rodovia Araraquara-Jaú, km. 01, Araraquara, 14800-903 São Paulo, Brazil; luciani.gaspar.toledo@gmail.com (L.G.D.T.); matheusramos_91@hotmail.com (M.A.D.S.R.); lari_sposito@hotmail.com (L.S.); fernandopavan@fcfar.unesp.br (F.R.P.); ericaoliveir@bol.com.br (É.D.O.L.); 2 Department of Infectious Diseases, Faculty of Medicine of São José do Rio Preto, São José do Rio Preto, 15090-000 São Paulo, Brazil; elza.maria.castilho@gmail.com (E.M.C.); margarete@famerp.br (M.T.G.D.A.); 3 Brazilian Agricultural Research Corporation, Embrapa Tropical Agroindustry, 60511-110 Fortaleza, Brazil; guilherme.zocolo@embrapa.br (G.J.Z.); alinynunes@outlook.com (F.A.N.S.); tigressa.rodrigues@embrapa.br (T.H.S.); 4 Department of Natural Active Principles and Toxicology, School of Pharmaceutical Sciences, Universidade Estadual Paulista, Araraquara, 14800-903 São Paulo, Brazil; santosag@fcfar.unesp.br

**Keywords:** *Cymbopogon nardus*, essential oil, gas chromatography, *Candida*, antifungal activity

## Abstract

**Background:** The incidence of fungal infections, especially those caused by *Candida* yeasts, has increased over the last two decades. However, the indicated therapy for fungal control has limitations. Hence, medicinal plants have emerged as an alternative in the search for new antifungal agents as they present compounds, such as essential oils, with important biological effects. Published data demonstrate important pharmacological properties of the essential oil of *Cymbopogon nardus* (L.) Rendle; these include anti-tumor, anti-nociceptive, and antibacterial activities, and so an investigation of this compound against pathogenic fungi is interesting. **Objective:** The aim of this study was to evaluate the chemical composition and biological potential of essential oil (EO) obtained from the leaves of *C. nardus* focusing on its antifungal profile against *Candida* species. **Methods:** The EO was obtained by hydrodistillation and analyzed by gas chromatography-mass spectrometry (GC-MS). Testing of the antifungal potential against standard and clinical strains was performed by determining the minimal inhibitory concentration (MIC), time-kill, inhibition of *Candida albicans* hyphae growth, and inhibition of mature biofilms. Additionally, the cytotoxicity was investigated by the IC_50_ against HepG-2 (hepatic) and MRC-5 (fibroblast) cell lines. **Results:** According to the chemical analysis, the main compounds of the EO were the oxygen-containing monoterpenes: citronellal, geranial, geraniol, citronellol, and neral. The results showed important antifungal potential for all strains tested with MIC values ranging from 250 to 1000 μg/mL, except for two clinical isolates of *C. tropicalis* (MIC > 1000 μg/mL). The time-kill assay showed that the EO inhibited the growth of the yeast and inhibited hyphal formation of *C. albicans* strains at concentrations ranging from 15.8 to 1000 μg/mL. Inhibition of mature biofilms of strains of *C. albicans*, *C. krusei* and *C. parapsilosis* occurred at a concentration of 10× MIC. The values of the IC_50_ for the EO were 96.6 μg/mL (HepG-2) and 33.1 μg/mL (MRC-5). **Conclusion:** As a major virulence mechanism is attributed to these types of infections, the EO is a promising compound to inhibit *Candida* species, especially considering its action against biofilm.

## 1. Introduction

*Candida* species have been a problem in human clinical practice due to the significant increase in cases of infection, especially in immunocompromised patients. The immune status of the host, the use of broad-spectrum antibiotics and corticosteroids, transplants, long-term intravascular and urethral catheters, and parenteral nutrition, are mentioned as risk factors in the development and increased incidence of fungal infections [1].

*Candida* species can develop on mucous membranes of the human body; this is associated with various types of diseases ranging from mucocutaneous overgrowth to disseminated infections [2]. Although the *C. albicans* is the prevalent species in candidemia, other species, such as *C. krusei*, *C. glabrata*, *C. tropicalis*, and *C. parapsilosis*, have been observed [3].

The ability of *Candida* species to cause disease is mainly related to mechanisms involving different virulence factors that include the morphological transition between yeast and hyphae, ability to defend themselves against the host immune system, adhesion, biofilm formation on host tissue or on medical devices, and production of harmful enzymes, such as hydrolytic proteases, phospholipases, and hemolysin [4].

Several antifungal agents have been indicated in the treatment of these infections, including those belonging to the polyenic, azole, and echinocandin classes; however, due to the indiscriminate use of these antimicrobial medications and physiological characteristics of the fungus, there has been a significant increase in resistance. Furthermore, the high toxicity, drug interactions, and insufficient bioavailability of active ingredients contribute to therapeutic failure [5].

Essential oils (EOs) from plants may be alternative bioactive compounds with antifungal properties because of the presence of secondary metabolites, such as tannins, terpenes, alkaloids, and flavonoids, etc. [6,7].

The genus *Cymbopogon* of the Poaceae family has been investigated for its pharmacological potential. *Cymbopogon nardus* (L.) Rendle, popularly known as citronella, is a grass cultivated in subtropical and tropical regions of Asia, Africa, and America, including Brazil [8]. The EO of the leaves of *C. nardus* is commonly used in perfumes, the production of cosmetics, and as an insect repellent. The major chemical constituents are geraniol, citral, citronellal, and citronellol [9]. Studies have demonstrated the antiviral [10], antibacterial [11], and antifungal activities [12] of this oil.

The EO is a complex mixture of monoterpene and sesquiterpene hydrocarbons (10 and 15 carbon atoms, respectively), and their oxygenated derivatives such as alcohols, aldehydes, and ketones, phenylpropanoids, and other minor compounds [13]. EOs are also called volatile oils or ethereal oils, as they have a high degree of evaporation when exposed to air at room temperature; this feature confers the strong odor to plants, both to attract pollinators and to repel insects and herbivores [14].

EOs are important in several areas of science, especially in combatting pathogenic or opportunistic microorganisms [15,16]. The presence of terpenes, as one of the chemical compounds in EO, contributes to the complex constitution with the action against microorganisms being directly related to this characteristic [17].

The antimicrobial potential demonstrated by terpenes (e.g., monoterpenes) is attributed to their interference in the integrity and functioning of the cell membrane through induction of changes in membrane potential, loss of cytoplasmic material and inhibition of the respiratory chain. Thus, these characteristics of EO are relevant in the search of new antifungal agents [18].

Considering the fungal etiology of different diseases with great impact on public health, and reports on the use of plants of the *Cymbopogon* genus in medical literature, the aim of this study was to evaluate the chemical composition and biological potential of the EO of *C. nardus*. This study focuses on the exploration of the antifungal profile against *Candida* species in order to present this compound as a possible antifungal or adjuvant agent.

## 2. Results and Discussion

### 2.1. Chemical Composition of Essential Oil

The qualitative and quantitative composition (GC-MS) of the EO is shown in Table 1. Oxygen-containing monoterpenes were the major constituents (90.61%), with citronellal (27.87%), geraniol (22.77%), geranial (14.54%), citronellol (11.85%), and neral (11.21%) as the main compounds. These monoterpenes are derived from geranyl diphosphate and are biosynthetically related [19].

The retention time (*t*_R_) of citronellal was 11.2 min by GC-FID analysis. The equation and *R*^2^ value obtained from the analytical curve for citronellal were *y* = 571,529.9016*x* − 102,555.3281 and 0.99976. The concentration of citronellal in the EO (GC-FID) was determined by means of the external standardization method as 282.5 mg/mL. Considering the relative density of commercial EO at 25 °C—0.897 g/mL (Sigma-Aldrich, St. Louis, MO, USA, 2016), the concentration of citronellal in the EO can be expressed as 31% (*m*/*m*). This value is consistent with the value obtained in the GC-MS analysis (28%).

In a study performed by Wei and Wee [22] the concentration of citronellal, the major compound (29.6%), was similar to this work. Koba et al. [23] and Trindade et al. [24] found higher concentrations of citronellal at 35.5% and 37.75%, respectively. In Thailand, the concentrations were different [13]: geraniol (35.7%), *trans*-citral (22.7%), *cis*-citral (14.2%), geranyl acetate (5.8%), citronellal (5.8%), and citronellol (4.6%). In another recent study, the authors also obtained a different chemical composition of EO with the main compounds being geraniol (25.9%), citronellal (3.7%), and citronellol (3.1%) [25].

### 2.2. Minimal Inhibitory Concentration and Minimal Fungicidal Concentration of Essential Oil of C. nardus

The antifungal activity of the EO is shown in Table 2. The solvent and growth controls presented satisfactory results. Thus, the antifungal activity was attributed to essential oil. The results show that the EO had effective antifungal activity with a MIC range of 250–1000 μg/mL, including for isolates resistant to fluconazole and amphotericin-B. The lowest MIC value (250 μg/mL) of the EO was seen against *C. krusei.* Furthermore, the EO showed fungicidal activity against all fungi except two clinical isolates of *C. tropicalis* that were resistant to the EO with MIC > 1000 μg/mL.

Unlike conventional antimicrobial drugs, the literature does not present a standard of MIC values (sensitive and resistant) for natural products against *Candida* species. A study performed by Webster et al. [26] that evaluated antifungal activity of 14 medicinal plant extracts, found MIC values equal to, or lower than, 1000 μg/mL. The authors believe that values equal to or lower than 1000 μg/mL confirm sensitivity.

The values observed in this study were satisfactory, as the EO exhibited inhibitory action against 90% of the strains tested. Although some strains were inhibited with the highest concentration evaluated (1000 μg/mL), these data are relevant, since most of the strains are resistant to fluconazole (MIC > 64 μg/mL), the main drug used in the medical practice [27].

Interestingly, the MIC values (500 to 250 μg/mL) of the EO against the *C. krusei* ATCC clinical isolate are promising, due to the fact that this species presents intrinsic resistance to azoles [28].

The study carried out by Nakahara et al. [12] demonstrated that the EO inhibited filamentous fungus from the environment, however, the methodology used to determine the MIC was different from this study.

Recent research by Trindade et al. [24] showed the antifungal activity of the EO against ATCC and clinical strains of *C. albicans* and *C. tropicalisi*, with MIC values ranging from 32 to 64 g/mL. The differences found are expected because factors, such as climate, region, and the time of harvest of *C. nardus*, in addition to the extraction method, can directly affect the characteristics and concentration of chemical compounds [29].

The assay used to determine the MFC showed that the fungicidal properties of EO against *Candida* species were capable of killing the fungal cells using the concentrations evaluated in this study.

The antifungal activity of terpenoids, one of the major groups of volatile secondary metabolites, is known in the pharmaceutical field [17]. Thus, the antifungal activity of the EO in this present study may be related to the monoterpenes identified in the GC-MS assay.

The anti-*Candida* potential of the terpenes, geraniol, and citronellol has been investigated previously, with effective inhibitory activity against *C. albicans* [18] and filamentous fungi of the *Aspergillus* species [30]. In addition, Mesa-Arango et al. [31] showed that oxygenated monoterpenes in the citral chemotype, such as geraniol, citral and citronellal, have antifungal activity against *C. parapsilosis*, *C. krusei*, *Aspergillus flavus*, and *Aspergillus fumigatus.*

### 2.3. Minimal Inhibitory Concentration and Minimal Fungicidal Concentration of Citronellal

The MIC and MFC of citronellal are shown in Table 3. Citronellal showed antifungal activity against *C. albicans* ATCC, *C. krusei* (ATCC and clinical strain), and *C. glabrata* (ATCC and clinical strain). The species *C. tropicalis*, *C. parapsilosis*, *C. orthopsilosis*, and *C. albicans* clinical strains were resistant to citronellal, with MIC > 1000 μg/mL. Thus, in this present investigation, EO had better antifungal activity compared to citronellal, probably owing to synergisms among the chemical compounds present in the EO.

### 2.4. Inhibition on Candida albicans Hyphae Growth

**Figure 1:**
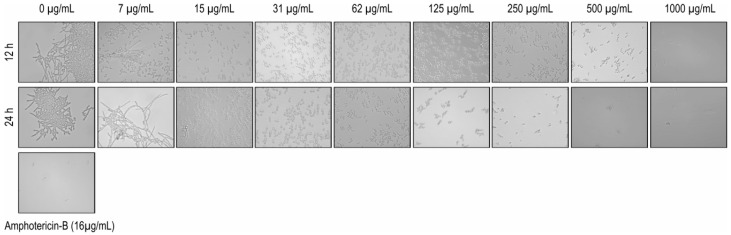
Inhibitory effect of essential oil of *C. nardus* on the transition of *C. albicans* from yeast to the hyphal form (photomicrographs by inverted light microscopic under 400× magnification).

The results exhibited that the EO was able to inhibit the transition of *C. albicans* from yeast to the hyphal form. Microscopic observation of EO-treated fungal cells revealed an absence of filamentous cells in concentrations ranging from 1000 to 15 μg/mL (after 12 and 24 h) (Figure 1).

Some therapeutic approaches are used to combat *C. albicans*, including blocking the transformation of yeast cells to filaments. This morphological change is considered to be a virulence factor, and, the biological mechanism has been explored using several active ingredients against this fungal species [32].

The ability of *C. albicans* to form hyphae is a risk factor in infections because hyphae play an important role in further tissue invasion due to their ability to adhere to host epithelial and endothelial cells [1]. Therefore, the results of this work are promising, since the EO was able to inhibit this morphological transition.

Leite et al. [33] showed the action of citral, a mixture of two geometric isomers known as neral and geranial, that are found at high concentrations in the EO used in this study. These authors found that these components were able to inhibit pseudohyphae, chlamydospores, and blastoconidia at the concentration of 128 μg/mL over 48 h.

The ability to EO to interfere in hyphal formation was demonstrated for different species of fungi. Chen et al., [8] for example, proved that the oil was able to promote deformities in the hyphal structure of the fungus *Alternaria.*

### 2.5. Time-Kill Assay

**Figure 2:**
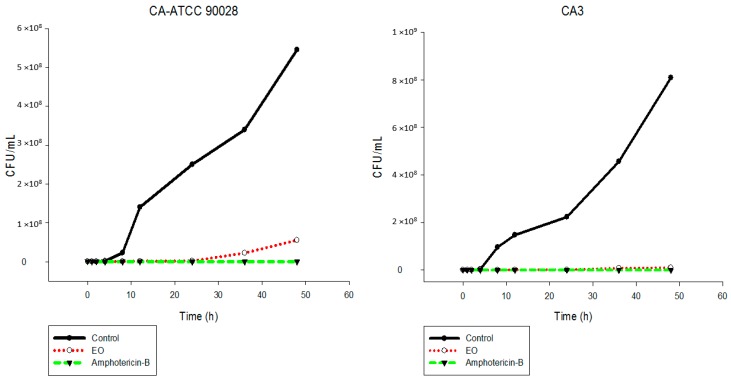
Time-kill curves of *C. albicans* ATCC 90028 and CA3 following exposure to the essential oil of *C. nardus* and amphotericin-B. Control represents the untreated *Candida* cells.

**Figure 3:**
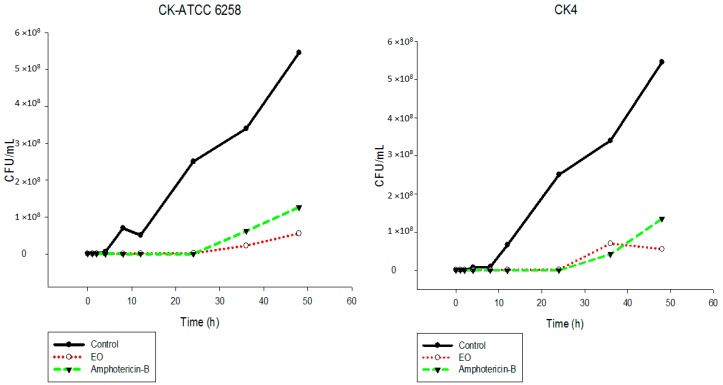
Time-kill curves of *C. krusei* ATCC 6258 and CK4 following exposure to the essential oil of *C. nardus* and amphotericin-B. Control represents the untreated *Candida* cells.

**Figure 4:**
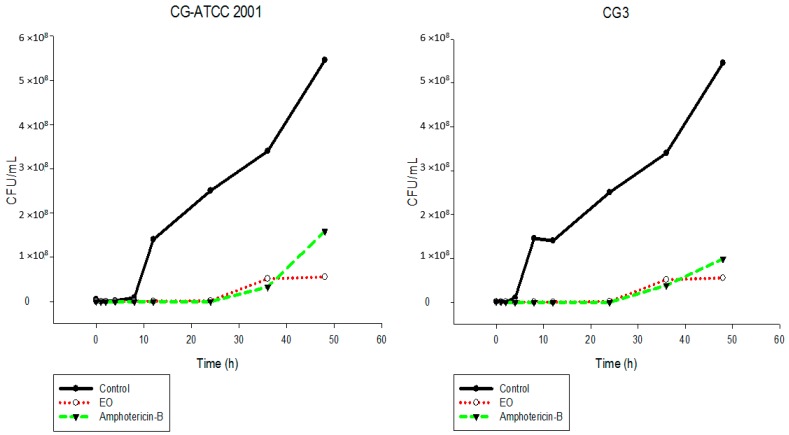
Time-kill curves of *C. glabrata* ATCC 2001 and CG3 following exposure to the essential oil of *C. nardus* and amphotericin-B. Control represents the untreated *Candida* cells.

**Figure 5:**
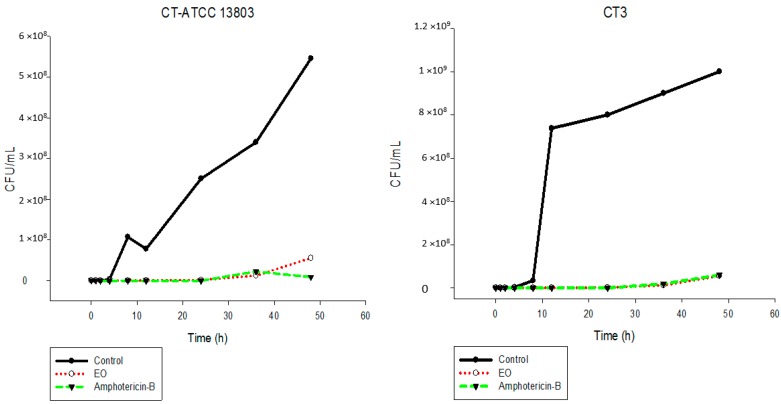
Time-kill curves of *C. tropicalis* ATCC 13803 and CT3 following exposure to the essential oil of *C. nardus* and amphotericin-B. Control represents the untreated *Candida* cells.

**Figure 6:**
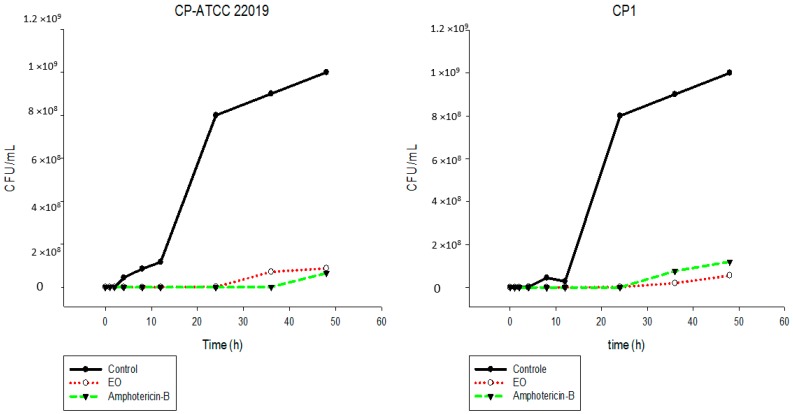
Time-kill curves of *C. parapsilosis* ATCC 22019 and CP1 following exposure to the essential oil of *C. nardus* and amphotericin-B. Control represents the untreated *Candida* cells.

**Figure 7:**
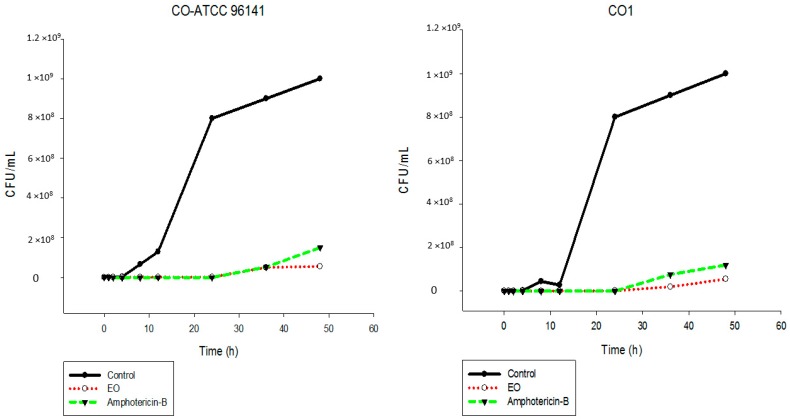
Time-kill curves of *C. orthopsilosis* ATCC 96141 and CO1 following exposure to the essential oil of *C. nardus* and amphotericin-B. Control represents the untreated *Candida* cells.

The results showed that the EO inhibited the fungal growth of the different *Candida* species in a similar manner (Figure 2, Figure 3, Figure 4, Figure 5, Figure 6 and Figure 7). The cell growth was constant until 24 h. After this, an exponential growth—CFU/mL—was noted which remained proportional for a long time. The strains CK-ATCC 6258, CK4, CG-ATCC 2001, GG3, CP1, CO-ATCC 96141, and CO1 presented a superior inhibitory behavior than amphotericin-B over 48 h.

The current literature does not present data on time-kill assays evaluating this EO against *Candida* species. Thus, this study has an innovator character. Ahmad and Viljon [25] observed synergic activity of EOs from the genus *Cymbopogon* with silver ions against fungi.

### 2.6. Biofilm

**Figure 8:**
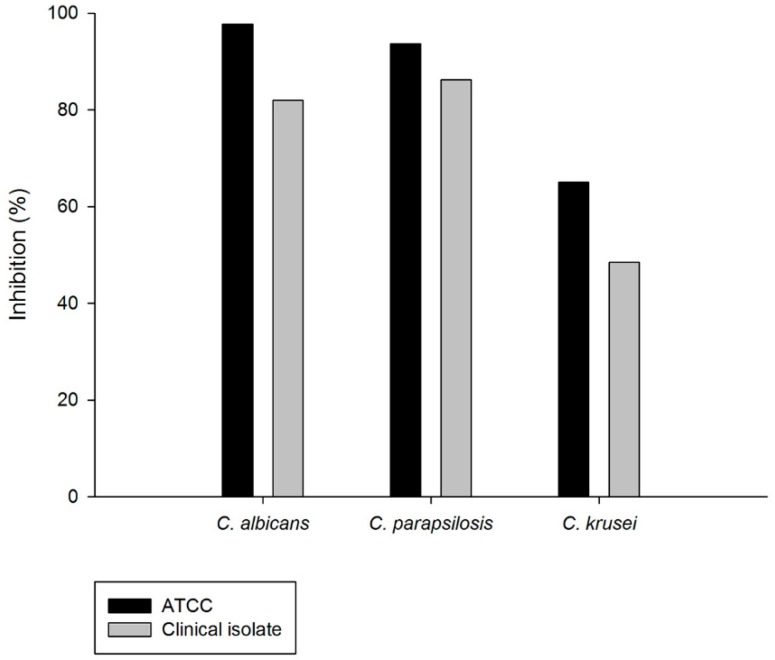
Percentage of inhibition of the essential oil of *C. nardus* against biofilms of *Candida* species.

Biofilms produced by *C. albicans*, *C. parapsilosis*, and *C. krusei* were treated with EO (10× MIC). The EO showed expressive anti-biofilm activity at different concentrations (Table 4). The percentage of inhibition of biofilms by EO is demonstrated in Figure 8. The EO showed high inhibition of biofilms, mainly against *C. albicans* ATCC (97.7%), CA3 (82.0%), *C. parapsilosis* ATCC (93.6%), CP1 (86.2%), *C. Krusei* ATCC (65.0%), and CK4 (48.5%).

The anti-biofilm potential of the EO was the main result of the present study. The elimination and control of fungal biofilm is very hard, due to several types of molecular, structural, and specifically physiological interactions [4]. Additionally, the mechanism of resistance presented by planktonic cells, and the quorum-sensing processes (signaling molecules), the production of specific enzymes and natural mutations can explain the increased resistance of biofilm to antimicrobial agents used in the clinical practice [1].

Biofilm is an important virulence factor and the treatment for its control is concentration dependent and can be high to inhibit the biofilm. The MIC to act against microbial biofilm ranges from 10–100 times higher than that necessary against the planktonic form [34].

The current results show that the EO was able to eliminate mature biofilm of *C. albicans* and *C. parapsilosis* at a concentration of 10× MIC. The scientific literature does not present any study of this EO against mature biofilm. Thus, these results are important and contribute to control strategies to eradicate mature biofilm.

### 2.7. Cytotoxic Evaluation

The concentrations of the EO and citronellal that inhibited cell vitality by 50% (IC_50_) are shown in Table 5. Citronellal demonstrated a higher IC_50_ than the EO. The EO exhibited inhibitory effects against HepG-2 and MRC-5 with IC_50_ values of 96.6 μg/mL and 33.1 μg/mL, respectively.

On comparing the results, the EO was less cytotoxic to HepG-2 than to MRC-5 cell lines. This can be explained by the metabolizer action of HepG-2. Although lower IC_50_ values were found, it is important to stress that amphotericin-B, the gold standard in the treatment of fungal diseases, has toxic effects, such as nephrotoxicity, and has an acute reaction after intravenous infusions [5]. In vivo tests must be performed because factors, such as immune response, metabolism, and the pharmacokinetics of the EO, are important.

## 3. Materials and Methods

### 3.1. Plant Material

The leaves of *C. nardus* (L.) Rendle were collected in July 2013, in the morning, from the Garden of Toxic and Medicinal Plants: “Profa. Dra. Célia Cebrian de Araújo Reis” (Universidade Estadual Paulista, Araraquara, São Paulo, Brazil). A voucher specimen (HRCB-60752) was deposited in the Rioclarense Herbarium of the Institute of Biosciences (Universidade Estadual Paulista, Rio Claro, São Paulo, Brazil).

### 3.2. Extraction of the Essential Oil from the Leaves of C. nardus

Fresh leaves of *C. nardus* (150 g) were submitted to hydrodistillation using a Clevenger-type apparatus attached to a round bottom flask (3 L) with 1500 mL of deionized water. The residual water in the EO was separated from the sample by freezing. The yield of the EO was 0, 7% (*w*/*w*). The EO was stored under refrigeration until chemical analysis and biological tests.

### 3.3. Citronellal

The commercial (+/−)− citronellal standard (≥95% purity) used was purchased from Sigma-Aldrich Co. (St. Louis, MO, USA).

### 3.4. Gas Chromatography Analysis of Essential Oil from the Leaves of C. nardus

#### 3.4.1. Gas Chromatography-Mass Spectrometry

Gas chromatography-mass spectrometry (GC-MS) analysis was performed using an Agilent^®^ GC-7890B/MSD-5977A gas chromatograph (mass detector: electron impact ionization; mass quadrupole analyzer, (Agilent^®^, Santa Clara, CA, USA) fitted with a HP-5ms capillary column 5% diphenyl-polydimethylsiloxane (30 m × 0.25 mm, film thickness 0.25 μm—Agilent^®^). Helium was used as the carrier gas at a flow rate of 1.00 mL/min (8.2 psi) and linear velocity of 36.6 cm/s. Injector temperature: 250 °C; injection volume: 1 μL; splitting ratio: 1:100; oven temperature program: 60–246 °C (3 °C/min, 62 min); transfer line temperature: 280 °C; detector temperature: 150 °C; and ionization energy: 70 eV. EO was solubilized in hexane (chromatographic grade; Tedia^®^, Fairfield, OH, USA) 1:100 (*v*/*v*). The identification of the EO components was based on the comparison of acquired mass spectra (from chromatogram peaks) with reference spectra of the NIST mass-spectral library version 2.0, 2012 (243,893 compounds) and data from the literature. Furthermore, arithmetic retention indices [20] were calculated as described in [21] by linear interpolation relative to the retention times (*t*_R_) of a series of *n*-alkanes (C7–C30); the obtained values were compared with published retention index values [21]. Relative amounts of EO components were calculated based on the chromatogram peak area normalization method.

#### 3.4.2. Gas Chromatography-Flame Ionization Detector

Gas chromatography-flame ionization detector (GC-FID) analysis was performed using a Shimadzu^®^ GC-2010 Plus gas chromatograph (flame ionization detector, Shimadzu^®^, Kyoto, Japan) fitted with a RTX-5MS capillary 5% diphenyl-polydimethylsiloxane column (30 m × 0.25 mm, film thickness 0.25 μm, Restek^®^, Bellefonte, PA, USA). Nitrogen was used as the carrier gas adjusted to a flow rate of 1.00 mL/min (8.2 psi) and linear velocity of 36.6 cm/s. Injector temperature: 250 °C; injection volume: 1 μL; splitting ratio: 1:30; oven temperature program: 70 °C–180 °C (4 °C/min) and 180 °C–250 °C (10 °C/min); total analysis time: 34.5 min; detector temperature: 280 °C. For the analytical curve, (+/−)− citronellal standard (Sigma-Aldrich^®^; ≥95% purity) solutions were prepared in hexane (chromatographic grade; Merck^®^): 1.0, 1.5, 2.0, 2.5, 3.0, 3.5, 4.0, and 5.0 mg/mL. EO was solubilized in hexane (chromatographic grade; Merck^®^) 1:100 (*v*/*v*). All analyses were performed in triplicate. The identification of citronellal in the EO was based on retention time (*t*_R_) and its quantification was achieved according to the external standard method using an analytical curve.

### 3.5. Antifungal Activity

### 3.6. Fungal Strains

The strains—20 samples of *Candida* spp.—were obtained from the Laboratory of Microbiology, Department of Infectious Diseases, Medicine School in Sao José do Rio Preto (FAMERP), São Paulo, Brazil. These included three clinical isolates and one ATCC for each species: *C. albicans* (CA-ATCC 90028, CA2, CA3, CA4); *C. krusei* (CK-ATCC 6258, CK2, CK3, CK4); *C. glabrata* (CG-ATCC 2001, CG2, CG3, CG4); *C. tropicalis* (CT-ATCC 13803, CT2, CT3, CT4), *parapsilosis* complex*—C. parapsilosis* (CP-ATCC 22019, CP1), and *C. orthopsilosis* (CO-ATCC 96141, CO1). *C. albicans* ATCC 10231 was used to test for inhibition of hyphal growth.

The clinical strains were donated to the Microbiology Laboratory of the Medicine School in Sao Jose do Rio Preto for the purposes of scientific research through a written consent of the donors. The use of these strains was approved by the Human Research Ethics Committee of FAMERP, project identification code 152/2006 (6 December2006), Medicine School in Sao José do Rio Preto (FAMERP).

### 3.7. Determination of Minimum Inhibitory Concentration

The evaluation of the antifungal activity by determining the MIC was performed by the microplate dilution technique according to the protocol described in the M27-A3 document [35] with modifications. The concentration of the EO and citronellal was 7.8 to 1000 μg/mL. The EO was dissolved in 10% methanol and 2% Tween 80. A quantity of 0.1 mL was placed in a 96-well microtiter plate containing Roswell Park Memorial Institute (RPMI) 1640 medium. Each well was inoculated with 0.1 mL of a suspension containing 2.5 × 10^3^ CFU/mL of yeast.

Amphotericin B (AmB) (Sigma-Aldrich^®^) and fluconazole (FLU) (Sigma-Aldrich^®^) were used as the positive controls. Additional controls also included the culture medium, yeast growth, EO, and solvent. The microplates were incubated at 37 °C for 48 h. After incubation, 20 μL of an aqueous 2% solution of 2,3,5-triphenyltetrazolium chloride (TTC) was added, the plates were incubated at 37 °C for 2 h [32], and absorbance of the samples was measured by spectrophotometer (Biospectro, SP22, Curitiba, Brazil). All tests were performed in triplicate.

According to obtained results of the EO MIC determination, the more sensitive strains (one ATCC and one clinical strain of each species) were selected to evaluate the antifungal activity of citronellal.

### 3.8. Determination of Minimum Fungicidal Concentration

An aliquot from each well that showed antifungal activity was plated in Petri dishes containing Sabouraud Dextrose Agar (SDA)—DIFCO, to determine the minimum fungicidal concentration (MFC). The assays were carried out in triplicate. MFC was defined as the lowest concentration of the EO and citronellal that allowed no visible growth on the solid medium [32].

### 3.9. Inhibition of C. albicans Hyphae Growth

A microassay was developed to evaluate the inhibition effect on the growth of fungal strains. Growth of *C. albicans* (ATCC 10231) from a 48 h culture was transferred to a microplate with RPMI 1640 medium supplemented with fetal bovine serum (FBS) to obtain a final concentration of 2.5 × 10^3^ yeast/mL. EO was added to the growth medium at concentrations ranging from 7.5 to 1000 μg/mL, and the cultures were incubated for 12 and 24 h at 37 °C. The hyphal formation of *C. albicans* was observed through an inverted light microscope. Amphotericin B (16 μg/mL) was used as a positive control [32].

### 3.10. Time-Kill Assay

The time-kill assay was performed according to Santos-Filho et al. [36], with modifications. This assay tested one ATCC strain and one clinical strain of each *Candida* species (CA ATCC 90028, CA3, CK ATCC 6258, CK4, CG ATCC 2001, CG3, CT ATCC 13803, CT3, CP ATCC 22019, CP1, CO ATCC, and CO1). In brief, Sabouraud Dextrose broth (SDB)-DIFCO, containing 2.5 × 10^3^ CFU/mL of *Candida* spp. and 2× MIC of EO were incubated at 37 °C and aliquots of 100 μL were removed at different time intervals (0, 1, 2, 4, 8, 12, 24, 36 and 48 h). The aliquots were then diluted in a buffer solution of sterile PBS 1:100, twice. Each EO-cell suspension was spread onto Sabouraud plates and colonies were counted after 48 h incubation at 37 °C. Amphotericin B was used as a positive control. Negative controls were established with cell suspensions without the addition of EO.

### 3.11. Biofilm Assay

The biofilm adhesion method was performed as described by Pitangui et al. [37], with modifications. The CA ATCC 90028, CA3, CK ATCC 6258, CK4, CP ATCC 22019, and CP1 strains were selected for the biofilm assay. Initially, 100 μL of inoculum (5.0 × 10^8^ cells/mL), suspended in 0.9% saline solution, was added to the wells of microplates (96 wells) and incubated in a shaker at 80 rpm at 37 °C for 2 h. After the pre-adhesion period, the supernatant was removed and 100 μL of RPMI medium was added to each microplate well. Incubation continued at 37 °C for 48 h with the RPMI renewed after 24 h. The supernatant was then removed, and the wells were washed with 100 μL of 0.9% saline solution. Next, 100 μL of EO (10× MIC) were added to each microplate well. The microplates were incubated again for 24 h at 37 °C. Subsequently, the EO was removed and the wells were washed with sterile saline solution (to eliminate the drug carryover effect). Solvent, medium culture, and yeast growth were established as controls with the colorimetric indicator 2,3-bis(2-methoxy-4-nitro-5-sulfophenyl)-5-[carbonyl(phenylamino)]-2*H*-tetrazolium hydroxide (XTT^®^, Sigma-Aldrich^®^, Saint Louis, MO, USA).

### 3.12. Cytotoxic Activity

#### 3.12.1. Cell Lines

HepG-2 (hepatic) (ATCC^®^HB-8065™, Fiocruz, Rio de Janeiro, Brazil) and MRC-5 (fibroblast) (ATCC^®^ CCl-171™, Fiocruz, Rio de Janeiro, Brazil) were used to determine cytotoxicity (IC_50_). The cells were maintained in flasks with a 12.50 cm^2^ surface area containing 10 mL of culture medium incubated at 37 °C in 5% carbon dioxide. The culture medium consisted of Dulbecco’s Modified Eagle Medium (DMEM, Vitrocell^®^, Campinas, São Paulo, Brazil) supplemented with 10% FBS, gentamicin sulfate (50 mg/L, Sigma-Aldrich^®^), and amphotericin B (2 mg/L, Sigma-Aldrich^®^).

#### 3.12.2. Cytotoxic Assay

The cytotoxic assay [38] consisted of collecting the cells using a solution of trypsin/ethylenediaminetetraacetic acid (EDTA, Vitrocell^®^), centrifuging the solution (2000 rpm for 5 min) and counting the number of cells in a Neubauer chamber followed by adjustment of the cell concentration to 7.5 × 10^4^ cells/mL in DMEM. Then, 200 μL of this suspension was placed in each well of a 96-well microplate to obtain a concentration of 1.5 × 10^4^ cells/well, and the microplates were then incubated at 37 °C in 5% carbon dioxide for 24 h to facilitate cell attachment to the plate. The serial dilutions of EO were prepared to obtain concentrations from 3.90 to 1000 μg/mL. These dilutions were added to the cells after the removal of the medium and the non-adherent cells. Then, the cells were incubated for an additional 24 h. The cytotoxicity of the compounds was determined by adding 30 μL of resazurin and reading on a microplate reader (BioTek^®^, Winoosky, VT, USA) after 6 h of incubation using a microplate and excitation emission filters with wavelengths of 530 and 590 nm, respectively. The IC_50_ was defined as the highest concentration of compound that allowed a viability of at least 50% of the cells. All experiments were performed in triplicate. A solution of 5% dimethyl sulfoxide (DMSO) was used as the control.

## 4. Conclusions

According to the results of this study, it is possible to conclude that the EO from *C. nardus* is a promising source of active molecules with antifungal properties. The biological assays reported in this investigation show that the EO inhibits ATCC and clinical strains of *Candida* species, including those with resistance to drugs employed in medical practice. Additional to this simple inhibitory activity, the EO is able to inhibit and control the main virulence factors attributed to the *Candida* species used in this study, such as the formation and proliferation of hyphae of *C. albicans* and, more importantly, the eradication of mature biofilms. Moreover, the EO exhibits better antifungal action than citronellal, probably due to some synergistic effect among the EO components.

## Figures and Tables

**Table 1:** Composition of essential oil from the leaves of *C. nardus*.

Retention Time	Compound Name	AI ^1^ (Calculated)	AI ^2^ (Literature)	Concentration (%)
6.60	*not identified*	–	–	0.09
6.74	β-myrcene	992	988	0.09
7.08	*n*-octanal	1004	998	0.09
7.92	D-limonene	1029	1024	2.47
8.21	*cis*-ocimene	1037	1032	0.27
8.56	*trans*-ocimene	1048	1044	0.17
8.75	bergamal	1053	1051	0.37
9.40	*not identified*	–	–	0.17
10.40	linalool	1101	1095	0.53
10.57	α-pinene oxide	1105	1099	0.11
11.51	*trans*-rose oxide	1129	1122	0.14
12.16	neo-isopulegol	1145	1144	0.41
12.34	*not identified*	–	–	0.27
12.54	citronellal	1155	1148	27.87
12.95	*not identified*	–	–	0.25
13.69	*not identified*	–	–	0.33
14.16	*cis*-4-decenal	1195	1193	0.09
14.63	Decanal	1207	1201	0.46
15.60	β-citronellol	1230	1223	11.85
16.12	Neral	1242	1235	11.21
16.73	geraniol	1257	1249	22.77
17.38	geranial	1273	1264	14.54
20.82	citronellol acetate	1355	1350	0.31
22.08	geranyl acetate	1385	1379	0.26
23.45	β-cariophyllene	1419	1417	1.28
24.81	α-humulene	1453	1452	0.12
27.25	γ-cadinene	1514	1513	1.60
27.63	δ-cadinene	1524	1522	0.36
27.87	citronellyl butyrate	1530	1530	0.24
28.60	elemol	1550	1548	0.11
29.11	*not identified*	–	–	0.16
29.85	cariophyllene oxide	1582	1582	0.55
32.08	*trans*-cadinol	1642	1638	0.16
32.54	α-muurolol	1654	1644	0.30
Monoterpene hydrocarbons				3.00
Oxygen containing monoterpenes				90.61
Sesquiterpene hydrocarbons				3.36
Oxygen containing sesquiterpenes				1.12
Other compounds				0.64
Total identified				98.73

^1^ Arithmetic retention indices [20] relative to C7-C30 *n*-alkanes calculated [21]; ^2^ Arithmetic retention indices [20,21].

**Table 2:** Inimal inhibitory concentrations (MIC—μg/mL) and minimal fungicidal concentration (MFC—μg/mL) of the essential oil of *C. nardus* against *Candida* species.

Candida Strains	MIC EO *	MFC EO *	MIC AmB *	MIC FLU *
CA-ATCC 90028	1000	1000	1	1
CA2	1000	1000	4	16
CA3	1000	1000	1	>64
CA4	1000	1000	4	8
CK-ATCC 6258	250	500	8	>64
CK2	500	500	8	>64
CK3	500	500	8	>64
CK4	250	250	4	>64
CG-ATCC 2001	500	1000	1	>64
CG2	500	1000	4	>64
CG3	500	1000	2	>64
CG4	1000	1000	2	>64
CT-ATCC 13803	500	1000	8	>64
CT2	>1000	>1000	8	>64
CT3	1000	>1000	4	>64
CT4	>1000	>1000	4	>64
CP-ATCC 22019	500	1000	4	8
CP1	1000	1000	4	32
CO-ATCC 96141	500	1000	8	32
CO1	1000	1000	8	64

FLU: fluconazole; AmB: Amphotericin-B; * values in μg/mL.

**Table 3:** Minimal inhibitory concentrations (MIC, μg/mL) and minimal fungicidal concentration (MFC, μg/mL) of citronellal against *Candida* species.

Strains	MIC Citronellal	MFC Citronellal
CA-ATCC 90028	1000	1000
CA3	>1000	>1000
CK-ATCC 6258	500	1000
CK4	500	500
CG-ATCC 2001	500	1000
CG3	500	>1000
CT-ATCC 13803	>1000	>1000
CT3	>1000	>1000
CP-ATCC 22019	>1000	>1000
CP1	>1000	>1000

**Table 4:** Anti-biofilm effect of the essential oil of *C. nardus* against *C. albicans*, *C. krusei*, and *C. parapsilosis*.

Strains	EO (mg/mL)
CA-ATCC 90028	2.5
CA3	5
CK-ATCC 6258	2.5
CK4	2.5
CP-ATCC 22019	5
CP1	10

**Table 5:** Cytotoxic activity of the essential oil of *C. nardus* (EO) and citronellal.

Cell Lines	(EO) IC_50_ *	Citronellal IC_50_ *	(Control) IC_50_^a^
HepG-2	96.6	100.9	>1000
MRC-5	33.1	51	>1000

* Values in μg/mL; ^a^ Dimethyl sulfoxide.
